# Mr. Stevenson, on Spurious Vaccine

**Published:** 1801-08

**Authors:** John Stevenson

**Affiliations:** Kegworth


### *To the Editors of the Medical and Phyfical Journal.*

GENTLEMEN,

IF the enclofed communication be deemed worthy a place in your excellent Mifcellany, its infertion will much oblige your conftant reader and well wifher,

*Kegworth, July* 1, 1801.

JOHN STEVENSON.

---

THE numerous and very refpectable teftimonials, detailed in the various numbers of your excellent Journal, relative to the fuccefsful and extenfive inoculation of the Vaccine Difeafe, might feem to render any further communications on the fubject unneceffary, fince its character as a prophylactic to the variolous infection is now, it may be fuppofed, fully eftablifhed. Notwithftanding, however, the mafs of *apparently satisfactory* evidence in its favor, I cannot perfuade myfelf that the practice has been brought to the zenith of perfection, and, as fuch, incapable of farther elucidation and improvement. Elfe, how

ſhall we reconcile the diſcordant repreſentations of its great ſeverity, and even mortality in ſome individuals, with the infinitely greater proportion of ſucceſsful caſes, in which the ſubjects of the diſeaſe have experienced little conſtitutional derangement, though they have equally participated of its ſalutary preventive influence? I am aware that the abettors of the new ſyſtem will anſwer, that this contrariety of effects depends upon the degenerated ſtate of the matter employed, and a want of diſcrimination between the genuine and ſpurious Vaccinia. It is not however to be expected, notwithſtanding the attention that has been beſtowed upon its inveſtigation by men eminently qualified both by their talents and opportunities for making accurate obſervations, that all its different modifications and nice dependencies are, at ſo early a period after its introduction, compleatly developed. I am ready to admit that where the Vaccine virus has exerted unuſual violence in its operation, the cauſe is to be ſought for in the peculiar irritability of the conſtitution of the individual at the time of inſertion, diſpoſing it to take on a morbid train of actions, rather than to its ſpecific deleterious nature. At the ſame time, from the two caſes which have fallen under my immediate inſpection, and which I ſhall here beg leave to enumerate, I do not feel perfectly ſatisfied that it is *univerſally* and *infallibly* an antidote to the Small-pox.

This ſuppoſition is at leaſt admiſſible, if we may be allowed to deduce our opinions from the prevalence of what are conſidered the diagnoſtic ſymptoms of the genuine Cow-pox, as in no inſtance that has come to my knowledge, has their exiſtance been ſo clearly marked, and ſo fully diveſted of obſcurity in every particular, as the following.

Maſter Thomas-Harvey-Toton Notts, aged two years, was inoculated with Vaccine matter on the 1ſt of June, 1800, taken from a young lady in the ſame village, on the ninth day of the diſeaſe, and immediatly inſerted without dilution, in its perfectly limpid ſtate, into both arms. On the fourth day, the matter had evidently taken effect, and on the fifth a ſmall veſicle appeared, which gradually enlarged till the eighth, when my patient became uneaſy, fretful, and ſomewhat feveriſh. Theſe ſymptoms abated in the ſpace of forty-eight hours. The beautiful eryſipelatous circumſcribed efflореſcence, or areola around the puſtule, which was diſtended with a tranſparent fluid, continued to increaſe in circumference till the twelfth day. From this period it ſpontaneouſly though gradually ſubſided, the matter in the puſtule being all this time of an aqueous colour and conſiſtence. The puſtule began now to aſſume a dark complexion, and an eſchar formed, which ſeparated in about about a fortnight afterwards. On the ninth day a few red eruptions appeared, fcattered thinly over the body like meafles, which in the fpace of four days turned brown, and foon defquamated, nor did they contain any fluid during their continuance. In fhort, I know not of any variation in the fymptoms from the commencement to the termination of the difeafe, from thofe which uniformly occur in the Cow-pox, fave the cutaneous eruptions, which are not a neceffary or ufual concomitant.

On the eighth day, Mafter Edward-Harvey, aged feven years, was inoculated with matter taken from his brother. It would be nugatory to ftate the particulars of his fymptoms, and it is only neceffary to mention that he went through the difeafe in a more mild, though equally diftinct form.

In fix months afterwards, both thefe young gentlemen were inoculated with recent variolous matter, in order to remove from the minds of their parents all doubts of the efficacy of the Cow-pox as a prefervative againft the contagion of the variola. As for myfelf, I entertained not the leaft apprehenfion of any effects from the inoculation, and in this conviction I deemed a preparative courfe quite fuperfluous. The matter which had been thus introduced, inftead of dying away on the third or fourth day as I had anticipated, began to produce inflammation on both their arms. You may conceive my confufion and chagrin, when on the eighth day, I received a meffage requefting me to vifit my young patients, who complained of head-ache, chillnefs, ficknefs, and the other precurfory fymptoms of Small-pox. On my arrival, I found, to my fincere regret, that there was little doubt of their having the genuine variolous fever. The puftules on the arms of both were fully diftended with purulent matter, and confiderably inflamed around their margins. In Mafter Edward, on the following day, a full crop of eruptions fupervened. With refpect to his brother, the eruptive fever was much milder, a circumftance that was owing probably to his being more expofed to the open air in addition to the very foluble ftate of his bowels. The puftules too never attained to that high degree of maturity as in Mafter Edward. For after being red, and bounded by a marginal inflammation, and being filled with a much lefs proportion of purulent contents, they fooner turned brown and exficcated, a fymptom not unufual in very favourable cafes of variola.

That this fecondary difeafe was the real Small-pox, admits not of a doubt, fince many children were inoculated fuccefsfully with matter taken from Mafter Edward. It may be proper to obferve in this place, that the young lady from whom Mafter T. Harvey received the Cow-pox, has been lately inoculated with variolous matter, but did not take the infection.

R 2 This

This fact coincides with some instances of Small pox, in which the patient (as is said on good authority), is endowed with the power of communicating the genuine disease to other individuals who shall be susceptible of the contagion a second time, whilst the constitution of the first is shielded from its repeated aggression.

These histories and reflections are transmitted, not with a view to depreciate this grand innovation in medicine, or to prejudice the minds of benevolent parents, anxious for the welfare of their tender offspring, against its further propagation. On the contrary, I feel sincerely disposed to congratulate the world at large on the happy introduction of the Jennerian Inoculation, from the conviction, that in various and important particulars it holds a decided preference over the Small-pox, which disease will probably, ere long, be completely exterminated, and known to future ages, probably, (as the true leprosy is to us), only by traditional knowledge. So far, therefore, am I from wishing to check the extending empire of its benign influence, that I publish the above cases, only as a corroboration of the close analogy that subsists in various relations between this new disease and the Small-pox. I am desirous also to stimulate those who have sufficient opportunities for the purpose, to endeavour to collect such characteristic features of the genuine idiopathic Cow-pox, as may enable practitioners to recognize, when it will prove certainly efficacious as a guardian to the constitution against the subsequent agency of the Variolous contagion, and under what circumstances a failure may, with equal probability, be apprehended.

This point appears the grand desideratum in the new Inoculation, which, if once obtained, would do away the mistakes arising from a spurious disease, and eventually establish its reputation on a permanent and unobjectionable basis.

